# Estimation of Vaccine Effectiveness of CoronaVac and BNT162b2 Against Severe Outcomes Over Time Among Patients With SARS-CoV-2 Omicron

**DOI:** 10.1001/jamanetworkopen.2022.54777

**Published:** 2023-02-03

**Authors:** Yuchen Wei, Katherine Min Jia, Shi Zhao, Chi Tim Hung, Chris Ka Pun Mok, Paul Kwok Ming Poon, Eman Yee Man Leung, Maggie Haitian Wang, Carrie Ho Kwan Yam, Tsz Yu Chow, Zihao Guo, Eng Kiong Yeoh, Ka Chun Chong

**Affiliations:** 1 School of Public Health and Primary Care, The Chinese University of Hong Kong, Hong Kong, China; 2 Center for Communicable Disease Dynamics, Department of Epidemiology, Harvard T.H. Chan School of Public Health, Boston, Massachusetts; 3 Li Ka Shing Institute of Health Sciences, Faculty of Medicine, The Chinese University of Hong Kong, Hong Kong, China

## Abstract

**Importance:**

Few studies have evaluated the waning of vaccine effectiveness against severe outcomes caused by SARS-CoV-2 Omicron infection. Hong Kong is providing inactivated and mRNA vaccines, but the population had limited protection from natural infections before the Omicron variant emerged.

**Objective:**

To examine the change in vaccine effectiveness against hospitalization and mortality due to the Omicron variant over time.

**Design, Setting, and Participants:**

This case-control study included adults with SARS-CoV-2 Omicron variant infection who died or were hospitalized in Hong Kong from January 1 to June 5, 2022 (ie, case participants), and adults with SARS-CoV-2 Omicron, sampled from the public health registry during the study period (ie, control participants), who were matched to case participants by propensity score.

**Exposures:**

Vaccination status of the individuals.

**Main Outcomes and Measures:**

Estimated vaccine effectiveness against death, death or hospitalization, and death among hospitalized patients. Vaccine effectiveness was calculated as 1 − adjusted odds ratio obtained by conditional logistic regression adjusted with covariates for each period following vaccination.

**Results:**

There were 32 823 case participants (25 546 [77.8%] ≥65 years; 16 930 [47.4%] female) and 131 328 control participants (100 041 [76.2%] ≥65 years; 66 625 [46.6%] female) in the sample analyzed for the death or hospitalization outcome. Vaccine effectiveness against death or hospitalization was maintained for at least 6 months after the second dose of both CoronaVac (74.0%; 95% CI, 71.8%-75.8%) and BNT162b2 (77.4%; 95% CI, 75.5%-79.0%) vaccines. Vaccine effectiveness against death in those aged 18 to 49 years was 86.4% (95% CI, 85.8%-87.0%) and 92.9% (95% CI, 92.6%-93.2%) for those receiving 2 doses of CoronaVac and BNT162b2, respectively, while for patients aged 80 years or older, it dropped to 61.4% (95% CI, 59.8%-63.2%) and 52.7% (95% CI, 50.2%-55.6%) for CoronaVac and BNT162b2, respectively. Nevertheless, overall vaccine effectiveness against death at 4 to 6 months after the third dose was greater than 90% for CoronaVac, BNT162b2, and the mixed vaccine schedule (eg, mixed vaccines: vaccine effectiveness, 92.2%; 95% CI, 89.2%-95.1%).

**Conclusions and Relevance:**

While vaccines were generally estimated to be effective against severe outcomes caused by SARS-CoV-2 Omicron infection, this analysis found that protection in older patients was more likely to wane 6 months after the second dose. Hence, a booster dose is recommended for older patients to restore immunity. This is especially critical in a setting like Hong Kong, where third-dose coverage is still insufficient among older residents.

## Key Points

**Question:**

Does vaccine effectiveness against hospitalization and mortality due to the SARS-CoV-2 Omicron variant change over time?

**Findings:**

In this case-control study with 164 151 participants, the CoronaVac and BNT162b2 vaccines were generally estimated to be effective against severe outcomes due to SARS-CoV-2 Omicron infection, but protection among older individuals was more likely to wane 6 months after the second dose.

**Meaning:**

These findings suggest that a booster dose should be recommended to older individuals to restore immunity, and this is especially urgent for a setting like Hong Kong, where third-dose coverage is still insufficient among older residents.

## Teaser

This case-control study examines change over time in estimated vaccine effectiveness against hospitalization and mortality caused by the SARS-CoV-2 Omicron variant among adults in Hong Kong.

## Introduction

The Omicron variant (B.1.1.529) of SARS-CoV-2 was first reported to the World Health Organization on November 24, 2021, from South Africa.^1^ With a rapid increase in infections in highly vaccinated populations, the efficacy of current vaccines against the Omicron variant remains a source of concern.^2,3,4,5^

Hong Kong had successfully contained the Delta variant spread, with almost no COVID-19 local cases for about 4 months before the Omicron variant emerged.^6,7,8^ Unfortunately, the rapidity and scale of the Omicron outbreak became uncontrollable in Hong Kong in early 2022. The daily COVID-19 death rate was among the highest ever recorded.^9,10^ In early 2021, the Hong Kong government authorized the free distribution of COVID-19 vaccines BNT162b2 mRNA vaccine (Pfizer-BioNTech) and CoronaVac inactivated vaccine (Sinovac) to the resident population. Approximately 85% of the population had received the second dose of the COVID-19 vaccine by June 5, 2022.^11^

To date, few studies have assessed the waning of protection against severe outcomes caused by SARS-CoV-2 Omicron infection. In this study, we used the official linked data to estimate how vaccine effectiveness (VE) against hospitalization and mortality changed over time after vaccination in patients with SARS-CoV-2 Omicron in Hong Kong, where the population had minimal protection against infections before the Omicron variant emerged.

## Methods

This is a case-control study using the linked administrative data. The Department of Health (DH) of the Government of the Hong Kong Special Administrative Region (HKSAR) permitted us to use the database in this investigation. All study data were completely anonymized. Ethics approval was obtained from the Survey and Behavioral Research Ethics Committee, The Chinese University of Hong Kong. Because this study was a retrospective analysis using secondary data with no personal information, the requirement for obtaining informed consent was waived. This study followed Strengthening the Reporting of Observational Studies in Epidemiology (STROBE) reporting guidelines for case-control studies.

### Data Sources

The data on registered patients with COVID-19 obtained from DH were linked to the vaccination database, which centralized population-based electronic vaccination records, including the date and type of each dose for Hong Kong residents who had been vaccinated. We also linked the hospital admissions database for patients with infection. The admission database was provided by the Hong Kong Hospital Authority, a statutory body managing all public hospitals in Hong Kong.

### Case-Control Design

The risk of hospitalization and death due to the SARS-CoV-2 Omicron variant was estimated using a case-control design in the vaccinated population vs the unvaccinated population. Data analysis was limited to an Omicron-predominant period spanning January 1 to June 5, 2022.

We used all registered patients with COVID-19 during the study period as our primary study population. Patients with COVID-19 aged 18 years or older with a positive result from a polymerase chain reaction (PCR) test or rapid antigen test (RAT) were included. Before mid-February 2022, the Omicron status of patients with PCR-confirmed COVID-19 was determined by genomic sequencing based on the defining mutations (N501Y and E484A). Due to laboratory capacity being overwhelmed by the rapid and massive increase in cases from no infections to more than 50 000 cases daily, the DH launched a self-registration system for PCR test and RAT results in mid-February 2022, a period dominated by the SARS-CoV-2 Omicron variant. While the variant status of all patients was not confirmed by genomic sequencing, subsampling revealed more than 99% of the specimens sequenced carried the mutated strain of the Omicron variant. Imported cases were excluded from the analysis. Aside from all registered patients, we included all patients aged 18 years or older who were hospitalized in public hospitals and were diagnosed with a COVID-19 infection by PCR test as a secondary study population.

### Study Outcomes

We used hospitalization or COVID-19 death, defined as the death episode with a positive test result, as a proxy for severe outcomes in the primary study population. The mortality data were corroborated by the birth and death records of HKSAR government, representing a complete coverage of deaths in Hong Kong. In the analysis of the hospitalization outcome, we excluded patients with COVID-19 who became infected during their hospital stay by excluding those whose PCR confirmation date was more than 3 days after admission. The data for this analysis did not include those admitted before week 6 in 2022; during that period, all confirmed cases were hospitalized regardless of clinical status following the government’s COVID-19 patient management policy. After the Omicron outbreak, the admission policy was changed, and only patients in severe or critical condition were admitted to hospitals.^12^ The number of days from the last dose (second or third dose) to the hospitalization or death date was used to calculate the time from vaccination to hospitalization or death. For the analyses of the hospitalized population, only COVID-19 death was used as the study outcome.

### Case and Control Groups

In the analysis of all the registered patients, case participants were the patients with COVID-19 who had the defined study outcomes (ie, hospitalization and death or death), and control participants were patients infected with SARS-CoV-2 who did not experience the study outcomes before the cutoff date. Because of the scarcity of individual-level data available for the community control population, case and control participants were only matched by age, sex, and the calendar date of COVID-19 infection.

In the analysis of the hospitalized patients, case participants were the patients with COVID-19 who had a positive PCR test on admission and died while hospitalized. Patients with SARS-CoV-2 who were hospitalized and alive before the cutoff date served as control participants. Given that clinical information could be linked to the hospitalization database, case and control participants were matched by the covariates of age, sex, chronic conditions or cancer, and use of oral COVID-19 treatment drugs (molnupiravir or paxlovid) or other coronavirus medications.

The propensity score method was performed for all case-control matches in a 1:4 ratio by using the nearest neighbor method without replacement, and the scores were determined by the logit link model regressing on the matching variables. We tested the robustness of the results by setting the matching ratio to 1:1 or 1:2.

### Exposure Assessment

Only individuals who received the BNT162b2 or CoronaVac vaccines were included in the analysis. Given the latency between vaccine ingestion and full development of immune responses, only a dose taken within 14 days of infection was considered a completed dose in the study.

### Statistical Analysis

The baseline characteristics of the case participants and matched control participants in the 2 study populations were descriptively compared. The adjusted odds ratio (AOR) for the association between vaccination and death was estimated using conditional logistic regression controlling for covariates. VE against the severe outcome was calculated as 1 − AOR. The 95% CIs of VE estimates were constructed by bootstrapping with 1000 resampled data sets with replacement. VE was compared across age groups (ie, 18-49, 50-64, 65-79, and ≥80 years), vaccine types, and number of doses. To assess the change in effectiveness over time, we estimated the VE of each vaccine for each period following vaccination (ie, 0-3, 4-6, and >6 months) using conditional logistic regression.

Vaccine protection over time, defined as the time from the last dose (second or third) to hospitalization or death for vaccinated individuals, was examined using the conditional Cox proportional hazard model adjusted for the covariates and is presented using the adjusted survival curves with an estimate of the median survival time of protection. Time from the last dose to infection was adjusted in the Cox model to control the temporal variation of coverage of the 2 vaccine types. A cutoff date of June 19, 2022, was used as the right censoring of the death outcome. R version 3.6 (R Project for Statistical Computing) was used for all the statistical analyses. Because this study used a large official administrative database with a high study power, no hypothesis testing was carried out.

## Results

**Figure 1.**
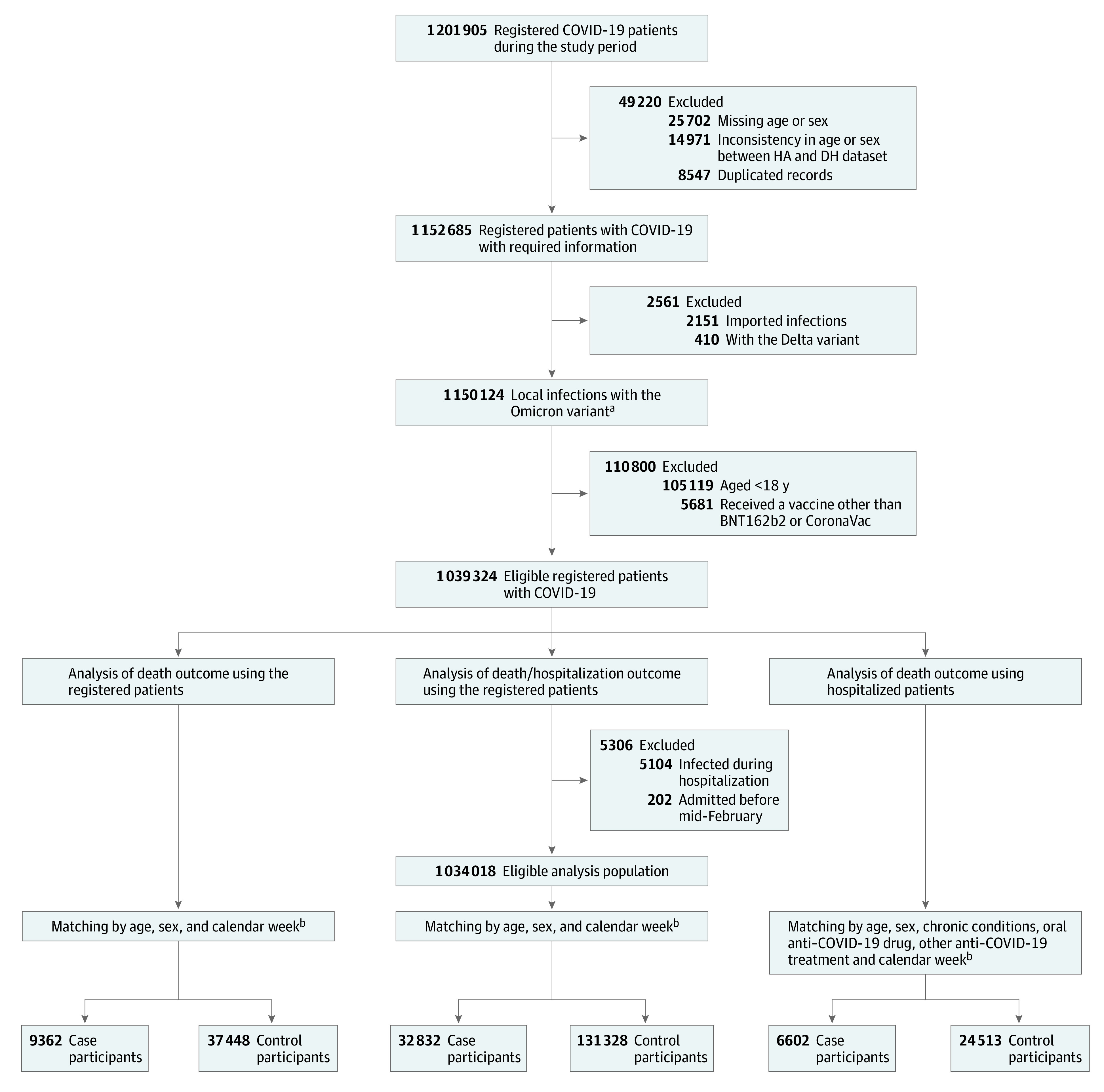
Procedure of Including Study Participants DH indicates Department of Health; HA, Hospital Authority. ^a^The Omicron variant status of patients with polymerase chain reaction–confirmed COVID-19 was determined by genomic sequencing based on the defining mutations (N501Y and E484A) before mid-February 2022. Due to laboratory capacity being overwhelmed by the rapid surge in cases, all confirmed cases using either polymerase chain reaction testing or rapid antigen tests were included after mid-February 2022, a period predominated by the SARS-CoV-2 Omicron variant. ^b^The propensity score method was conducted for all case-control matches in a 1:4 ratio, and the scores were determined based on the available covariates.

In all registered patients, 9362 deaths and 37 488 matched controls were included in the analysis of death outcome, whereas 32 832 case participants with death or hospitalization and 131 328 matched controls were included in the analysis of the death or hospitalization outcome (Figure 1). In the hospitalized population, 6602 deaths and 24 513 matched controls were included in the analysis of death outcome. To control for confounding by exposure risk during the Omicron epidemic, case and control participants were matched by calendar week (eFigure 1 in Supplement 1).

The characteristics of the case and control participants were generally balanced within each of the matching groups (Table 1). In the case group (n = 32 832) of the 164 160 registered patients in the analysis of the hospitalization or death outcome, 3149 (9.6%), 4137 (12.6%), 9104 (27.7%), and 16 442 (50.1%) were aged 18 to 49, 50 to 64, 65 to 79, and 80 years or older respectively, whereas in the control population (n = 131 328), 12 924 (9.8%), 18 363 (14.0%), 58 656 (44.7%), and 41 385 (31.5%) were aged 18 to 49, 50 to 64, 65 to 79, and 80 years or older, respectively. In this group, 16 930 case participants (47.4%) and 66 624 control participants (46.6%) were female patients. In the case group (n = 6602) of the 31 115 hospitalized patients, 64 (1.0%), 342 (5.2%), 1412 (21.4%), and 4784 (72.5%) were aged 18 to 49, 50 to 64, 65 to 79, and 80 years or older respectively, whereas, in the control population (n = 24 513), 2792 (11.4%), 3281 (13.4%), 6808 (27.8%), and 11 632 (47.5%) were aged 18 to 49, 50 to 64, 65 to79, and 80 years or older respectively. Among hospitalized case participants, 2746 (41.6%) and 3856 (58.4%) were female and male patients, respectively, whereas among the control participants, 11 875 (48.4%) and 12 638 (51.6%) were female and male patients, respectively.

**Table 1.** Characteristics of Cases and Controls

Characteristic	No. (%)					
	All registered patients^a^				Hospitalized patients, death outcome^b^	
	Death outcome		Hospitalization or death outcome		Case group (n = 6602)	Control group (n = 24 513)
	Case group (n = 9362)	Control group (n = 37 448)	Case group (n = 32 832)	Control group (n = 131 328)		
Age, y						
18-49	104 (1.1)	416 (1.1)	3149 (9.6)	12 924 (9.8)	64 (1.0)	2792 (11.4)
50-64	580 (6.2)	2320 (6.2)	4137 (12.6)	18 363 (14.0)	342 (5.2)	3281 (13.4)
65-79	2087 (22.3)	8347 (22.3)	9104 (27.7)	58 656 (44.7)	1412 (21.4)	6808 (27.8)
≥80	6591 (70.4)	26 365 (70.4)	16 442 (50.1)	41 385 (31.5)	4784 (72.5)	11 632 (47.5)
Sex						
Female	3834 (41.0)	15 291 (40.8)	16 930 (47.4)	66 625 (46.6)	2746 (41.6)	11 875 (48.4)
Male	5528 (59.0)	22 157 (59.2)	18 810 (52.6)	76 335 (53.4)	3856 (58.4)	12 638 (51.6)
Vaccination status						
Unvaccinated	5813 (62.1)	13 838 (37.0)	16 236 (49.5)	28 124 (21.4)	4605 (69.8)	11 435 (46.6)
1 Dose of BNT162b2	170 (1.8)	889 (2.4)	863 (2.6)	3907 (3.0)	110 (1.7)	695 (2.8)
1 Dose of CoronaVac	1282 (13.7)	6501 (17.4)	5460 (16.6)	19 765 (15.1)	1047 (15.9)	4271 (17.4)
2 Doses of BNT162b2	730 (7.8)	4724 (12.6)	3068 (9.3)	23 151 (17.6)	151 (2.3)	2479 (10.1)
2 Doses of CoronaVac	1121 (12.0)	8132 (21.7)	5317 (16.2)	33 528 (25.5)	613 (9.3)	4238 (17.3)
3 Doses of BNT162b2	79 (0.8)	922 (2.5)	640 (1.9)	7166 (5.5)	16 (0.2)	474 (1.9)
3 Doses of CoronaVac	137 (1.5)	2031 (5.4)	1032 (3.1)	12 728 (9.7)	49 (0.7)	768 (3.1)
Mixed doses (ie, 2 doses of CoronaVac with 1 dose of BNT162b2)	30 (0.3)	411 (1.1)	216 (0.7)	2959 (2.3)	11 (0.2)	153 (0.6)
Detection methods						
PCR testing	8827 (94.3)	24 884 (66.4)	28 624 (87.2)	77 134 (58.7)	6067 (91.9)	20 559 (83.9)
RAT	535 (5.7)	12 564 (33.6)	4208 (12.8)	54 194 (41.3)	535 (8.1)	3954 (16.1)
Chronic conditions or cancer						
Hypertension	NA	NA	NA	NA	2211 (33.5)	8454 (34.5)
Diabetes	NA	NA	NA	NA	1276 (19.3)	4790 (19.5)
Other cardiovascular diseases	NA	NA	NA	NA	1703 (25.8)	6532 (26.6)
Cancer	NA	NA	NA	NA	481 (7.3)	1706 (7.0)
Chronic lung disease	NA	NA	NA	NA	416 (6.3)	1444 (5.9)
Chronic kidney disease	NA	NA	NA	NA	500 (7.6)	1884 (7.7)
Chronic liver disease	NA	NA	NA	NA	336 (5.1)	1308 (5.3)
Obesity	NA	NA	NA	NA	206 (3.1)	865 (3.5)
Inpatient treatment received						
Molnupiravir	NA	NA	NA	NA	540 (8.2)	2799 (11.4)
Paxlovid	NA	NA	NA	NA	137 (2.1)	920 (3.8)
Remdesivir	NA	NA	NA	NA	1162 (17.6)	2080 (8.5)
Subcutaneous interferon β-1b	NA	NA	NA	NA	318 (4.8)	421 (1.7)
Dexamethasone	NA	NA	NA	NA	4684 (70.9)	6823 (27.8)
Intravenous tocilizumub	NA	NA	NA	NA	157 (2.4)	123 (0.5)

Abbreviations: NA, not applicable; PCR, polymerase chain reaction; RAT, rapid antigen test.

^a^ All registered patients included those aged 18 years or older who were diagnosed with a positive PCR test or RAT during the study period.

^b^ Hospitalized patients included all local patients aged 18 years or older who were hospitalized and diagnosed with Omicron infection by genomic sequencing. Their hospitalization records were linked with the test results to provide details on comorbidities and inpatient treatment received.

In the registered population, most case participants were unvaccinated (5813 [62.1%] in the death outcome group and 16 236 [49.5%] in the death or hospitalization outcome group), while only 13 838 control participants (37.0%) in the death outcome and 28 124 (21.4%) in the death or hospitalization outcome were unvaccinated. Table 2 displays estimated VE by vaccine type and number of doses in the 2 study populations. As anticipated, VE increased with the number doses for both types of vaccines. In the primary population, the VE of CoronaVac against death increased from 56.4% (95% CI, 54.7%-58.0%) at the first dose to 71.1% (95% CI, 69.8%-72.2%) at the second dose and to 88.5% (95% CI, 87.4%-89.6%) at the third dose, whereas that of BNT162b2 increased from 62.2% (95% CI, 58.5%-65.7%) at the first dose to 70.8% (95% CI, 69.0%-72.4%) at the second dose and to 84.4% (95% CI, 82.3%-86.2%) at the third dose. The VE of CoronaVac against hospitalization or death increased from 47.4% (95% CI, 44.5%-49.9%) at the first dose to 70.6% (95% CI, 68.8%-72.3%) at the second dose, and to 84.5% (95% CI, 82.4%-86.2%) at the third dose, whereas that of BNT162b2 increased from 58.0% (95% CI, 52.9%-61.8%) at the first dose to 76.8% (95% CI, 74.9%-78.3%) at the second dose and to 85.8% (95% CI, 83.3%-88.3%) at the third dose. Mixing doses of CoronaVac and BNT162b2 were as effective against hospitalization or death (VE, 85.1%; 95% CI, 83.7%-86.4%) and death (VE, 87.3%; 95% CI, 84.6%-89.4%) as the other 2 types of vaccine. Varying the case-control matching ratio did not strongly affect the estimates of VE (eTables 1 and 2 in Supplement 1).

**Table 2.** Estimated VE Against Severe Outcomes of SARS-CoV-2 Omicron Infection by Vaccine Type, Number of Doses, and Time After Dose

Vaccine type and time since last dose	All registered patients^a^				Hospitalized patients^a^	
	No. of hospitalizations/total No.	No. of deaths/total No.	VE against hospitalization or death (95% CI)^b^	VE against death (95% CI)^b^	No. of deaths/total No.	VE against death (95% CI)^b^
**1 Dose**						
CoronaVac	5290/25 225	1282/7783	47.4 (44.5-49.9)	56.4 (54.7-58.0)	1047/5318	28.3 (25.2-31.3)
BNT162b2	812/4770	170/1059	58.0 (52.9-61.8)	62.2 (58.5-65.7)	110/805	32.1 (23.6-40.3)
**2 Doses**						
CoronaVac						
Overall	4858/38 845	1121/9253	70.6 (68.8-72.3)	71.1 (69.8-72.2)	613/4851	51.0 (48.5-54.3)
Within 0-3 mo	2225/15 667	398/3825	69.1 (67.4-71.3)	74.7 (73.1-76.1)	287/1996	54.1 (51.1-57.9)
Within 4-6 mo	1390/11 127	389/2907	71.2 (69.1-73.3)	70.3 (68.5-71.9)	213/1504	43.0 (37.9-47.7)
>6 mo	1243/12 052	334/2521	74.0 (71.8-75.8)	70.5 (68.4-72.4)	113/1351	55.5 (49.7-60.4)
BNT162b2						
Overall	2506/26 219	730/5454	76.8 (74.9-78.3)	70.8 (69.0-72.4)	151/2630	54.5 (49.8-59.2)
Within 0-3 mo	671/5603	80/1132	73.9 (70.7-76.6)	85.5 (83.5-87.2)	50/620	56.2 (48.3-62.7)
Within 4-6 mo	658/7182	223/1738	76.4 (73.8-78.4)	71.3 (69.2-73.7)	42/749	56.9 (49.1-64.0)
>6 mo	1177/13 434	427/2583	77.4 (75.5-79.0)	62.6 (59.7-64.7)	59/1261	59.3 (52.4-66.1)
**3 Doses**						
CoronaVac						
Overall	948/13 760	137/2168	84.5 (82.4-86.2)	88.5 (87.4-89.6)	49/817	75.9 (71.4-80.0)
Within 0-3 mo	717/9836	108/1568	84.3 (82.5-85.8)	87.4 (86.0-88.7)	35/624	74.7 (69.5-79.1)
Within 4-6 mo	229/3902	29/600	87.7 (85.7-89.8)	91.6 (90.0-93.2)	14/192	83.0 (75.4-89.1)
BNT162b2						
Overall	577/7806	79/1001	85.8 (83.3-88.3)	84.4 (82.3-86.2)	16/490	78.9 (72.3-84.0)
Within 0-3 mo	485/6515	71/847	84.4 (82.3-85.9)	83.1 (81.1-85.2)	13/431	77.6 (69.3-84.4)
Within 4-6 mo	88/1279	8/154	84.7 (79.8-88.6)	90.2 (86.2-93.7)	3/58	71.5 (53.5-83.8)
Mixed						
Overall	197/3175	30/441	85.1 (83.7-86.4)	87.3 (84.6-89.4)	11/164	64.5 (49.8-76.1)
Within 0-3 mo	138/2055	23/275	85.9 (82.9-88.6)	84.9 (81.1-88.0)	10/107	Undefined^c^
Within 4-6 mo	59/1116	7/166	86.4 (82.3-90.0)	92.2 (89.2-95.1)	1/57	Undefined^c^

Abbreviation: VE, vaccine effectiveness.

^a^ There were 5813 deaths among 19 651 unvaccinated patients and 15 304 hospitalizations among 44 360 unvaccinated patients in all registered patients. There were 4605 deaths among 16 040 unvaccinated patients in the hospitalized group.

^b^ VE was calculated as 1 − the adjusted odds ratio, which was obtained using a conditional logistic regression model adjusted with the covariates. The unvaccinated group was used as the reference level compared with the vaccinated group in a specific subgroup (ie, vaccine type and time after dose). Time after dose (in months) is defined as the time since the last dose (second or third) to hospitalization or death for vaccinated individuals. One thousand bootstrapped samples were used to construct the 95% CI around the mean estimates.

^c^ The estimates cannot be drawn due to insufficient size of outcome.

**Figure 2.**
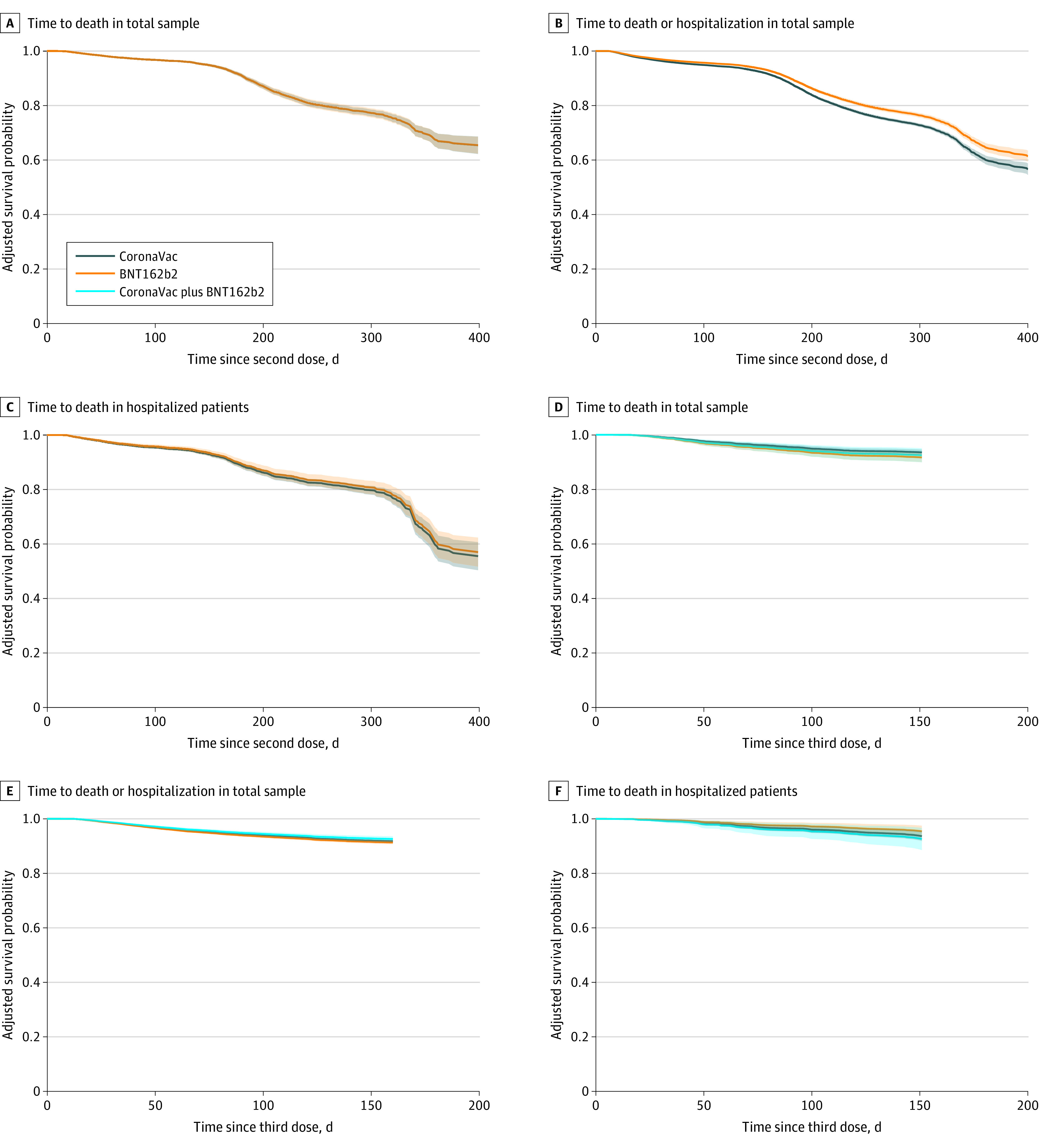
Survival Distribution of Time Since the Second and Third Doses of Vaccination to an Event by Vaccine Type The survival probability was determined by using the conditional Cox proportional hazard model adjusted with the covariates. Time from vaccination to an event was calculated as the number of days between the date of the last dose and the date of an event. The shaded areas represent 95% CIs.

Two doses of CoronaVac and BNT162b2 had similar VE estimates (Figure 2). The VEs against hospitalization or death (CoronaVac: VE, 74.0%; 95% CI, 71.8%-75.8%; BNT162b2: VE, 77.4%; 95% CI, 75.5%-79.0%) and death (CoronaVac: VE, 70.5%; 95% CI, 68.4%-72.4%; BNT162b2: VE, 62.6; 95% CI, 59.7%-64.7%) were preserved at 6 months and longer after the last dose in all registered patients (Table 2). The changes in VE of the 3 doses over time were similar across vaccine types (Figure 2). Specifically, the VE of CoronaVac, BNT162b2, and the mixed doses against death at 4 to 6 months after the third dose was 91.6% (95% CI, 90.0%-93.2%), 90.2% (95% CI, 86.2%-93.7%), and 92.2% (95% CI, 89.2%-95.1%), respectively.

**Figure 3.**
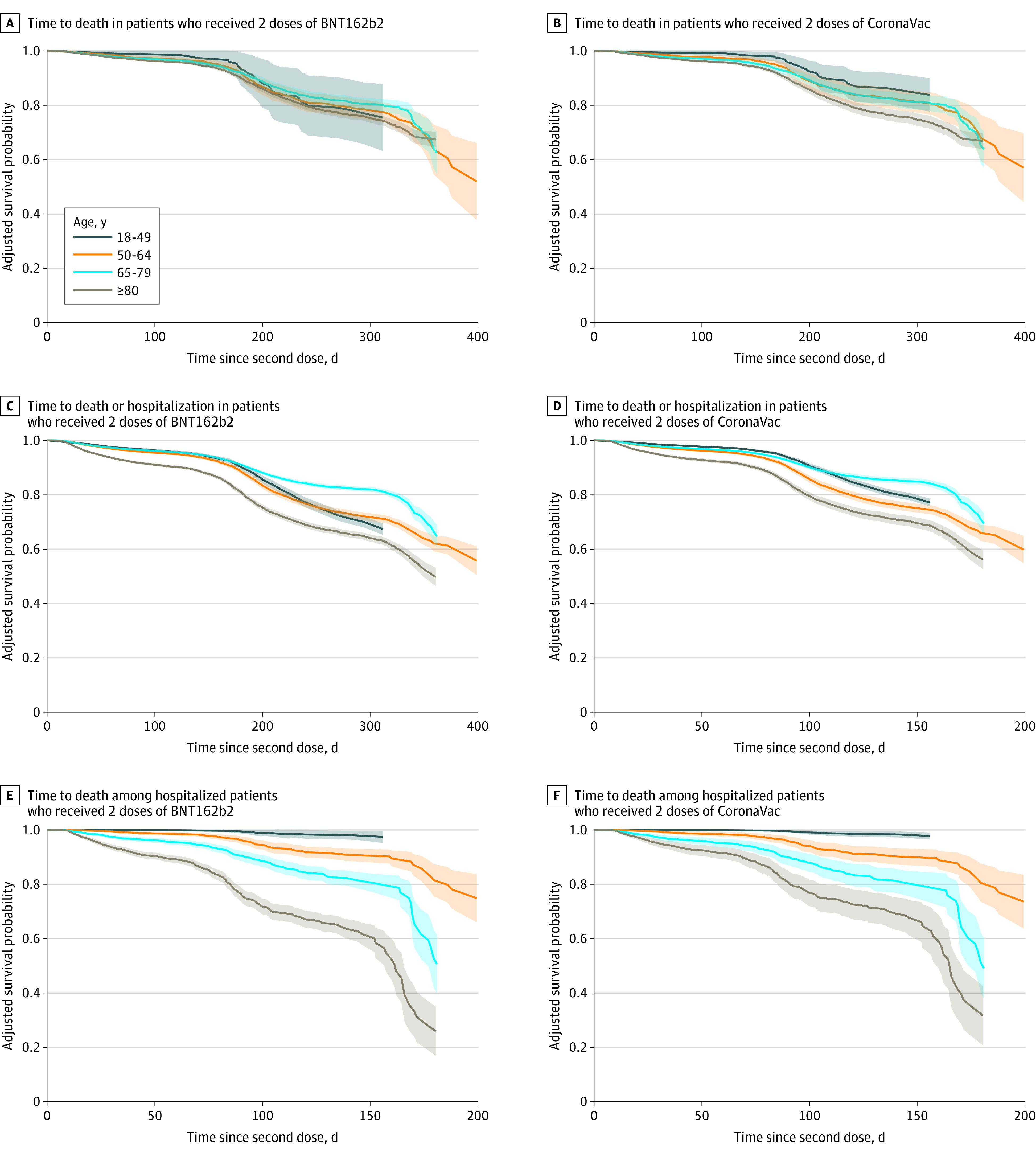
Survival Distribution of Time After the Second Dose of Vaccination to an Event by Age Group The survival probability was determined by using the conditional Cox proportional hazard model adjusted with the covariates. Time since vaccination to an event was calculated as the number of days between the date of the second dose and the date of an event. The shaded areas represent 95% CIs.

Across age groups, both types of vaccines were less effective in older groups (eFigure 2 in Supplement 1). In registered patients, VE against death in patients aged 18 to 49 years was 86.4% (95% CI, 85.8%-87.0%) and 92.9% (95% CI, 92.6%-93.2%) for those receiving 2 doses of CoronaVac and BNT162b2, respectively, while for patients aged 80 years or older, it lowered to 61.4% (95% CI, 59.8%-63.2%) and 52.7% (95% CI, 50.2%-55.6%), respectively. Furthermore, there was a pronounced decline in VE for older individuals in the hospitalized population even after controlling for covariates (Figure 3). The median survival time of the protection against death for 2 doses of CoronaVac and BNT162b2 in hospitalized patients aged 80 years or older was 323 days (95% CI, 313-330 days) and 330 days (95% CI, 315-341 days), respectively. Nonetheless, vaccine effectiveness for a 3-dose schedule remained greater than 90.0% for all vaccine types in the total sample (eg, mixed vaccines: vaccine effectiveness, 92.2%; 95% CI, 89.2%-95.1%), greater than 80.0% for all vaccine types in each age group, and there was no discernible difference in the decline of vaccine protection following the third dose across age groups (eFigure 3 in Supplement 1).

## Discussion

The sudden influx of infections caused by the Omicron variant in early 2022 overwhelmed the health system and depleted critical care resources in Hong Kong. In this study using linked administrative data, we found that the VE of 2 or 3 doses of mRNA or inactivated vaccine was estimated to remain durable over time. The results are generally consistent with other studies,^13,14,15,16^ including one in Qatar where the VE of BNT162b2 against severe Omicron outcomes was estimated to be 77.5% in 7 months after 2 doses and 90.1% in 7 weeks after 3 doses.^13^ Similarly, a study from England found that the VE of mRNA vaccines against severe outcomes caused by Omicron infection was greater than 80% 14 days after the second dose in patients aged 18 to 64 years and greater than 90% 7 to 104 days after the booster dose in patients aged 65 years and older.^14^

Our study assessed VE in the hospitalized population. In a cohort study of hospitalized older patients with COVID-19 infections during the Omicron wave in Israel, 3 doses of BNT162b2 did not provide a significant protection from death or mechanical ventilation compared with no vaccination.^17^ Their finding is inconsistent with our study, likely due to a comparatively longer time after the third dose in that population, provided that the waning postdose protection of BNT162b2 was similar to what we observed in our study. Another single-center study used a population with positive COVID-19 PCR test on hospital admission and found that the VE of 2 or more doses of Sputnik V (Gamaleya Research Centre) against severe and critical conditions remained greater than 90%.^18^ The inclusion of patients with the Delta variant and receiving a different vaccine type in their study affects the compatibility of their findings with ours.

While several studies have found that CoronaVac was less effective against severe outcomes than BNT162b2,^19,20^ our study suggested that both CoronaVac and BNT162b2 provide protection against severe outcomes caused by the Omicron variant and the VE waned consistently after the second dose. In fact, a national study in Chile found that CoronaVac was 86% effective against death in early 2021, when the Gamma and Alpha variants were circulating.^21^ In comparison with the antibody titer developed after 2 doses of BNT162b2, which decreased by 4.7-fold within 5 to 6 months, the antibody titer developed after CoronaVac reduced by 6- to 10-fold (depending on dosage and interval) within 6 months,^22,23^ potentially indicating a lower efficacy in protection.^24^ Nonetheless, vaccine recipients retained cellular immunity that protected against progression to severe COVID-19 even after neutralizing antibodies waned.^2^

According to our findings, a booster dose offered durable protection against severe outcomes caused by the Omicron variant over time. While several studies have documented the benefit of the BNT162b2 booster,^13,25^ very few empirical studies have investigated boosting in CoronaVac recipients, including those who received heterologous boosting (2 doses of CoronaVac followed by 1 BNT162b2 booster).^26^ While neutralization tests revealed that binding and neutralizing antibodies were higher after heterologous boosters than after homologous boosters,^27^ we were unable to demonstrate that a heterologous booster was more effective than 3 doses of CoronaVac. Nonetheless, our findings were consistent with those of a Chilean cohort study suggesting that either 3 doses of CoronaVac or a heterologous booster after a primary series of CoronaVac could result in a VE greater than 85% against death.^28^

Our research found that VE was lower in older patients, and protection was more likely to wane after 6 months following the second dose. This finding echoed a study in Finland showing that VE against hospitalization 6 months after 2 doses of BNT162b2 was only 61% in older patients during the Omicron-dominant period.^29^ It also echoed an early Hong Kong study that found VE was lower in older patients, even when they were fully vaccinated.^30^ Nevertheless, our findings suggest that the booster dose was capable of restoring VE and maintaining protection over time. Indeed, neutralization tests among individuals who received BNT162b2 revealed that a third dose of the vaccine increased antibody titer by more than 7-fold compared with the level after the second dose in individuals aged 65 to 85 years.^22^ The third dose of CoronaVac administered 8 months after the primary series completion increased titers by 3- to 5-fold compared with the level after 2 doses. Antibodies increased in both age groups younger and older than 60 years, with titers slightly higher in the older group.^23^

### Limitations

Our study has several major limitations. First, although local public health officials launched a self-registration system for reporting RAT results during the rapidly rising phase of the Omicron epidemic, the database would have missed a certain number of cases with mild symptoms or asymptomatic cases due to underreporting and other factors. Second, because critical care facilities were overcrowded during the peak of infections, many patients were unable to receive intensive care support; thus, the number of patients receiving care in intensive care units is an unreliable proxy for disease severity. Therefore, we excluded this indicator when defining disease severity, which inevitably resulted in inconsistency when generalizing the findings to other studies. Third, other studies found that seroconversion rates and antibody titers following COVID-19 vaccines were low in both immunocompromised and immunocompetent patients.^31^ Because our study was a retrospective secondary analysis, immunocompromise status was unavailable in the linked administrative database, which may have affected VE estimates. Fourth, because we had limited information on other covariates in the self-report system, we were unable to adjust for confounders other than age and sex. The analysis could also have been confounded by behavioral factors (eg, health care–seeking behavior and use of face masks) and lifestyle factors (eg, smoking and alcohol drinking). Nevertheless, we were able to link hospitalization records to account for chronic conditions and inpatient treatments in the analyses of hospitalized population.

## Conclusions

In this case-control study, we found that vaccines had high estimated VE against severe outcomes in patients with SARS-CoV-2 Omicron, but that protection in older patients was more likely to wane 6 months after the second dose. Therefore, a booster dose is recommended for older individuals to restore immunity. This is especially critical in a setting like Hong Kong, where coverage of the third dose of the vaccine is still insufficient among older residents.

## Notes

Supplement 1. **eFigure 1.** Matching of Cases and Controls by Number of Doses and Study Week **eFigure 2. **Vaccine Effectiveness Against Severe Outcomes of SARS-CoV-2 Omicron Infection by Age Group **eFigure 3. **Survival Distribution of Time Since the Third Dose of Vaccination to an Event by Age Group **eTable 1.** Distribution of Age in Cases and Controls When Setting Case-Control Matching Ratio at 1:1 and 1:2 **eTable 2.** Vaccine Effectiveness Against Severe Outcomes by Vaccine Type, Number of Doses, Time After Dose, and Case-Control Matching Ratios

Supplement 2.
Data Sharing Statement

## References

[1] World Health Organization. Classification of Omicron (B.1.1.529): SARS-CoV-2 variant of concern. November 2, 2021. Accessed January 3, 2023. https://www.who.int/news/item/26-11-2021-classification-of-omicron-(b.1.1.529)-sars-cov-2-variant-of-concern

[2] Andrews N, Stowe J, Kirsebom F, . COVID-19 vaccine effectiveness against the Omicron (B.1.1.529) variant. N Engl J Med. 2022;386(16):1532-1546. doi:10.1056/NEJMoa2119451 35249272 PMC8908811

[3] Collie S, Champion J, Moultrie H, Bekker LG, Gray G. Effectiveness of BNT162b2 vaccine against Omicron variant in South Africa. N Engl J Med. 2022;386(5):494-496. doi:10.1056/NEJMc2119270 34965358 PMC8757569

[4] Lopez Bernal J, Andrews N, Gower C, . Effectiveness of the Pfizer-BioNTech and Oxford-AstraZeneca vaccines on COVID-19 related symptoms, hospital admissions, and mortality in older adults in England: *test* negative case-control study. BMJ. 2021;373:n1088. doi:10.1136/bmj.n1088 33985964 PMC8116636

[5] Lauring AS, Tenforde MW, Chappell JD, ; Influenza and Other Viruses in the Acutely Ill (IVY) Network. Clinical severity of, and effectiveness of mRNA vaccines against, COVID-19 from Omicron, Delta, and Alpha SARS-CoV-2 variants in the United States: prospective observational study. BMJ. 2022;376:e069761. doi:10.1136/bmj-2021-069761 35264324 PMC8905308

[6] Lai CKC, Ng RWY, Wong MCS, . Epidemiological characteristics of the first 100 cases of coronavirus disease 2019 (COVID-19) in Hong Kong Special Administrative Region, China, a city with a stringent containment policy. Int J Epidemiol. 2020;49(4):1096-1105. doi:10.1093/ije/dyaa106 32601677 PMC7337784

[7] Wong MCS, Ng RWY, Chong KC, . Stringent containment measures without complete city lockdown to achieve low incidence and mortality across two waves of COVID-19 in Hong Kong. BMJ Glob Health. 2020;5(10):e003573. doi:10.1136/bmjgh-2020-003573 33028700 PMC7542625

[8] Yeoh EK, Chong KC, Chiew CJ, . Assessing the impact of non-pharmaceutical interventions on the transmissibility and severity of COVID-19 during the first five months in the Western Pacific Region. One Health. 2021;12:100213. doi:10.1016/j.onehlt.2021.100213 33506086 PMC7816004

[9] Cheung PH, Chan CP, Jin DY. Lessons learned from the fifth wave of COVID-19 in Hong Kong in early 2022. Emerg Microbes Infect. 2022;11(1):1072-1078. doi:10.1080/22221751.2022.2060137 35348429 PMC9004509

[10] Ma A, Parry J. When Hong Kong’s “dynamic zero” COVID-19 strategy met Omicron, low vaccination rates sent deaths soaring. BMJ. 2022;377:o980. doi:10.1136/bmj.o980 35418475

[11] Hong Kong Government. Hong Kong vaccination dashboard. Accessed January 3, 2023. https://www.covidvaccine.gov.hk/en/dashboard

[12] Hong Kong Government. Multi-tiered triage measures for treatment and isolation. Accessed January 3, 2023. https://www.coronavirus.gov.hk/pdf/tp_tiers.pdf

[13] Chemaitelly H, Ayoub HH, AlMukdad S, . Duration of mRNA vaccine protection against SARS-CoV-2 Omicron BA.1 and BA.2 subvariants in Qatar. Nat Commun. 2022;13(1):3082. doi:10.1038/s41467-022-30895-3 35654888 PMC9163167

[14] Stowe J, Andrews N, Kirsebom F, Ramsay M, Bernal JL. Effectiveness of COVID-19 vaccines against Omicron and Delta hospitalisation, a test negative case-control study. Nat Commun. 2022;13(1):5736. doi:10.1038/s41467-022-33378-7 36180428 PMC9523190

[15] Wright BJ, Tideman S, Diaz GA, French T, Parsons GT, Robicsek A. Comparative vaccine effectiveness against severe COVID-19 over time in US hospital administrative data: a case-control study. Lancet Respir Med. 2022;10(6):557-565. doi:10.1016/S2213-2600(22)00042-X 35227415 PMC8881000

[16] Tenforde MW, Self WH, Gaglani M, ; IVY Network. Effectiveness of mRNA vaccination in preventing COVID-19-associated invasive mechanical ventilation and death—United States, March 2021-January 2022. MMWR Morb Mortal Wkly Rep. 2022;71(12):459-465. doi:10.15585/mmwr.mm7112e1 35324878 PMC8956334

[17] Brosh-Nissimov T, Hussein K, Wiener-Well Y, . Hospitalized patients with severe COVID-19 during the Omicron wave in Israel—benefits of a fourth vaccine dose. Clin Infect Dis. Published online June 20, 2022. doi:10.1093/cid/ciac501 35724127 PMC9278185

[18] Shkoda AS, Gushchin VA, Ogarkova DA, . Sputnik V effectiveness against hospitalization with COVID-19 during Omicron dominance. Vaccines (Basel). 2022;10(6):938. doi:10.3390/vaccines10060938 35746546 PMC9227631

[19] Zheng C, Shao W, Chen X, Zhang B, Wang G, Zhang W. Real-world effectiveness of COVID-19 vaccines: a literature review and meta-analysis. Int J Infect Dis. 2022;114:252-260. doi:10.1016/j.ijid.2021.11.009 34800687 PMC8595975

[20] Suah JL, Husin M, Tok PSK, . Waning COVID-19 vaccine effectiveness for BNT162b2 and CoronaVac in Malaysia: an observational study. Int J Infect Dis. 2022;119:69-76. doi:10.1016/j.ijid.2022.03.028 35331933 PMC8938298

[21] Jara A, Undurraga EA, González C, . Effectiveness of an inactivated SARS-CoV-2 vaccine in Chile. N Engl J Med. 2021;385(10):875-884. doi:10.1056/NEJMoa2107715 34233097 PMC8279092

[22] Levin EG, Lustig Y, Cohen C, . Waning immune humoral response to BNT162b2 COVID-19 vaccine over 6 months. N Engl J Med. 2021;385(24):e84. doi:10.1056/NEJMoa2114583 34614326 PMC8522797

[23] Zeng G, Wu Q, Pan H, . Immunogenicity and safety of a third dose of CoronaVac, and immune persistence of a two-dose schedule, in healthy adults: interim results from two single-centre, double-blind, randomised, placebo-controlled phase 2 clinical trials. Lancet Infect Dis. 2022;22(4):483-495. doi:10.1016/S1473-3099(21)00681-2 34890537 PMC8651254

[24] Cromer D, Steain M, Reynaldi A, . Neutralising antibody titres as predictors of protection against SARS-CoV-2 variants and the impact of boosting: a meta-analysis. Lancet Microbe. 2022;3(1):e52-e61. doi:10.1016/S2666-5247(21)00267-6 34806056 PMC8592563

[25] Abu-Raddad LJ, Chemaitelly H, Ayoub HH, . Effect of mRNA vaccine boosters against SARS-CoV-2 Omicron infection in Qatar. N Engl J Med. 2022;386(19):1804-1816. doi:10.1056/NEJMoa2200797 35263534 PMC8929389

[26] Feikin DR, Abu-Raddad LJ, Andrews N, . Assessing vaccine effectiveness against severe COVID-19 disease caused by Omicron variant: report from a meeting of the World Health Organization. Vaccine. 2022;40(26):3516-3527. doi:10.1016/j.vaccine.2022.04.069 35595662 PMC9058052

[27] Costa Clemens SA, Weckx L, Clemens R, ; RHH-001 study team. Heterologous versus homologous COVID-19 booster vaccination in previous recipients of two doses of CoronaVac COVID-19 vaccine in Brazil (RHH-001): a phase 4, non-inferiority, single blind, randomised study. Lancet. 2022;399(10324):521-529. doi:10.1016/S0140-6736(22)00094-0 35074136 PMC8782575

[28] Jara A, Undurraga EA, Zubizarreta JR, . Effectiveness of homologous and heterologous booster doses for an inactivated SARS-CoV-2 vaccine: a large-scale prospective cohort study. Lancet Glob Health. 2022;10(6):e798-e806. doi:10.1016/S2214-109X(22)00112-7 35472300 PMC9034854

[29] Baum U, Poukka E, Leino T, Kilpi T, Nohynek H, Palmu AA. High vaccine effectiveness against severe COVID-19 in the elderly in Finland before and after the emergence of Omicron. BMC Infect Dis. 2022;22(1):816. doi:10.1186/s12879-022-07814-4 36335289 PMC9636823

[30] McMenamin ME, Nealon J, Lin Y, . Vaccine effectiveness of one, two, and three doses of BNT162b2 and CoronaVac against COVID-19 in Hong Kong: a population-based observational study. Lancet Infect Dis. 2022;22(10):1435-1443. doi:10.1016/S1473-3099(22)00345-0 35850128 PMC9286709

[31] Lee ARYB, Wong SY, Chai LYA, . Efficacy of COVID-19 vaccines in immunocompromised patients: systematic review and meta-analysis. BMJ. 2022;376:e068632. doi:10.1136/bmj-2021-068632 35236664 PMC8889026

