# Supplementary material for: Estimation of Vaccine Effectiveness of CoronaVac and BNT162b2 Against Severe Outcomes Over Time Among Patients With SARS-CoV-2 Omicron
Source: JAMA Netw Open. 2023 Feb 3;6(2):e2254777. doi: 10.1001/jamanetworkopen.2022.54777 (PMC9898822; doi:10.1001/jamanetworkopen.2022.54777)
Supplement: Supplement 1. — eFigure 1. Matching of Cases and Controls by Number of Doses and Study Week eFigure 2. Vaccine Effectiveness Against Severe Outcomes of SARS-CoV-2 Omicron Infection by Age Group eFigure 3. Survival Distribution of Time Since the Third Dose of Vaccination to an Event by Age Group eTable 1. Distribution of Age in Cases and Controls When Setting Case-Control Matching Ratio at 1:1 and 1:2 eTable 2. Vaccine Effectiveness Against Severe Outcomes by Vaccine Type, Number of Doses, Time After Dose, and Case-Control Matching Ratios [file jamanetwopen-e2254777-s001.pdf]

Supplemental Online Content

Wei Y, Jia KM, Zhao S, et al. Estimation of vaccine effectiveness of CoronaVac and BNT162b2 against severe outcomes over time among patients with SARS-CoV-2 Omicron. *JAMA Netw Open.* 2023;6(2):e2254777. doi:10.1001/jamanetworkopen.2022.54777

eFigure 1. Matching of Cases and Controls by Number of Doses and Study Week

eFigure 2. Vaccine Effectiveness Against Severe Outcomes of SARS-CoV-2 Omicron Infection by Age Group

eFigure 3. Survival Distribution of Time Since the Third Dose of Vaccination to an Event by Age Group

eTable 1. Distribution of Age in Cases and Controls When Setting Case-Control Matching Ratio at 1:1 and 1:2

eTable 2. Vaccine Effectiveness Against Severe Outcomes by Vaccine Type, Number of Doses, Time After Dose, and Case-Control Matching Ratios

This supplemental material has been provided by the authors to give readers additional information about their work.

© 2023 Wei Y et al. *JAMA Network Open.*

eFigure 1. Matching of Cases and Controls by Number of Doses and Study Week

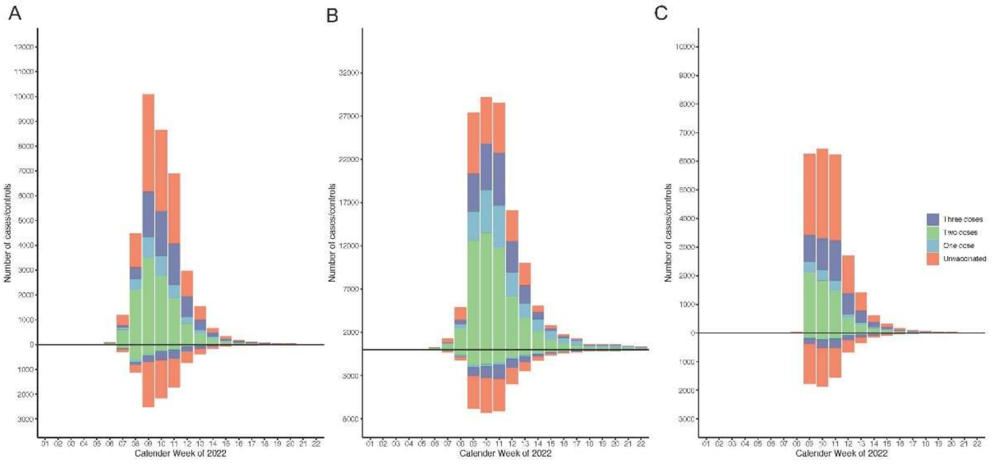

(A) Outcomes of deaths, (B) hospitalization/deaths in all registered patients; (C) Outcomes of death in hospitalized patients. Cases were defined as the infected patients who experienced the defined study outcome, and the controls were the infected patients without experiencing the study outcomes before the cut-off date. The cases and matched controls are presented in the lower and upper panel in each of the figures.

© 2023 Wei Y et al. *JAMA Network Open.*

eFigure 2. Vaccine Effectiveness Against Severe Outcomes of SARS-CoV-2 Omicron Infection by Age Group

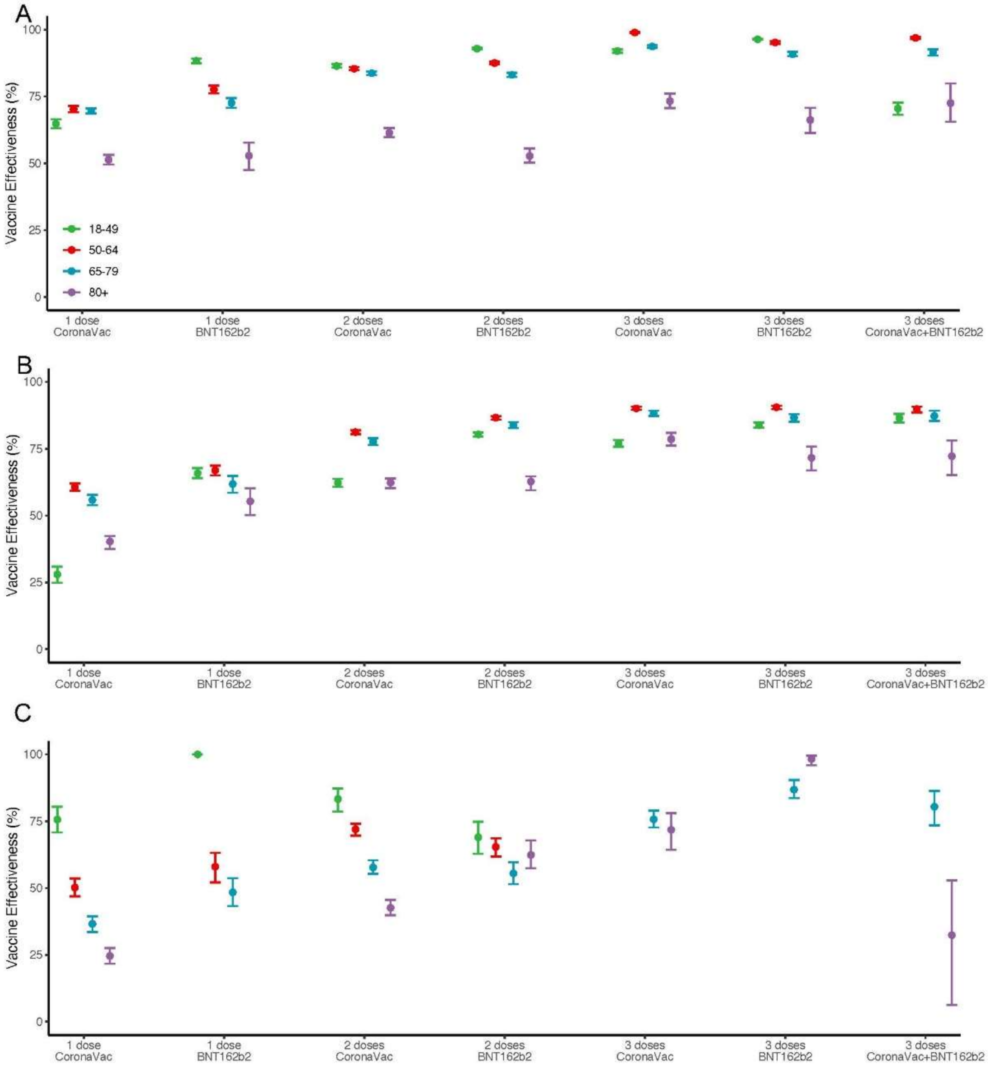

(A) Deaths in all registered patients; (B) Hospitalization/deaths in all registered patients; (C) Death in hospitalized patients. Vaccination effectiveness was calculated as 1 minus the adjusted odds ratio which was obtained using a conditional logistic regression model adjusted with the covariates. Unvaccinated group was used as the reference level. The error bars represent 95% confidence intervals constructed by bootstrapping with 1,000 resampled datasets with replacement.

© 2023 Wei Y et al. *JAMA Network Open.*

eFigure 3. Survival Distribution of Time Since the Third Dose of Vaccination to an Event by Age Group

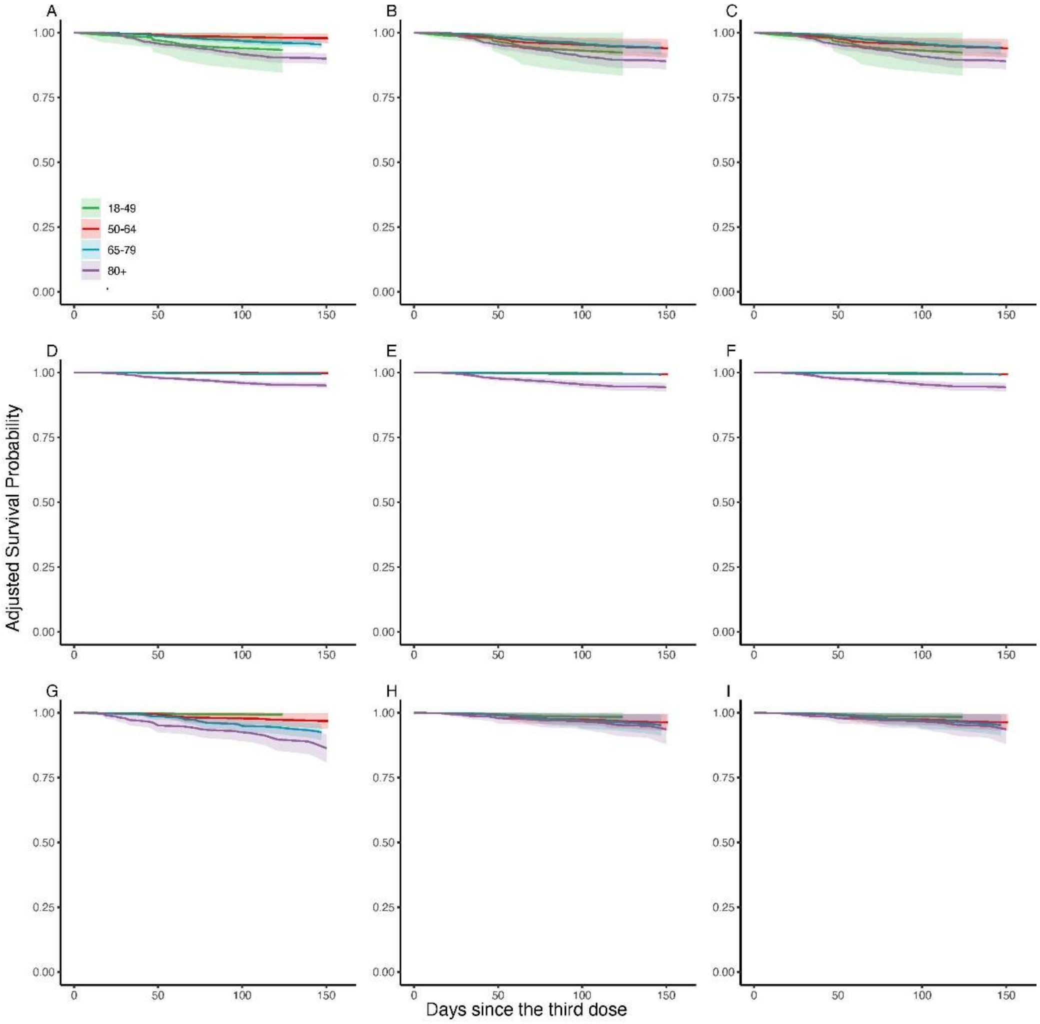

(A)-(C) Time since the third dose of vaccination to deaths in all registered patients receiving CoronaVac, BNT162b2, and mixing doses (two doses of CoronaVac + one dose of BNT162b2) respectively; (D)-(F) Time since the third dose of vaccination to hospitalization/deaths in all registered patients receiving CoronaVac, BNT162b2, and mixing doses respectively; (G)-(I) Time since the third dose of vaccination to deaths in hospitalized patients receiving CoronaVac, BNT162b2, and mixing doses respectively. The survival probability was determined by using the conditional Cox proportional hazard model adjusted with the covariates. Time since vaccination to an event was calculated as the number of days between the date of the third dose and the date of an event. The shaded areas represent 95% confidence intervals.

© 2023 Wei Y et al. *JAMA Network Open.*

eTable 1. Distribution of Age in Cases and Controls When Setting Case-Control Matching Ratio at 1:1 and 1:2

	All registered patients		Hospitalized patients	
	Hospitalization/death outcome		Death outcome	
Matching ratio at 1:1	Cases (n= 32,832)	Controls (n= 32,832)	Cases (n= 6,602)	Controls (n= 6,602)
Age, years				
18-49	3,149 (9.6%)	3149 (9.6%)	64 (1.0%)	51 (0.8%)
50-64	4,137 (12.6%)	4,137 (12.6%)	342 (5.2%)	345 (5.2%)
65-79	9,104 (27.7%)	9,110 (27.7%)	1,412 (21.4%)	1,557 (23.6%)
≥80	16,442 (50.1%)	16,436 (50.1%)	4,784 (72.5%)	4,649 (70.4%)
Matching ratio at 1:2	Cases (n= 32,832)	Controls (n= 65,664)	Cases (n= 6,602)	Controls (n= 13,204)
Age, years				
18-49	3,149 (9.6%)	6,298 (9.6%)	64 (1.0%)	159 (1.2%)
50-64	4,137 (12.6%)	8,293 (12.6%)	342 (5.2%)	763 (5.8%)
65-79	9,104 (27.7%)	19,880 (30.3%)	1,412 (21.4%)	3,821 (28.9%)
≥80	16,442 (50.1%)	31,193 (47.5%)	4,784 (72.5%)	8,461 (64.1%)

© 2023 Wei Y et al. *JAMA Network Open.*

eTable 2. Vaccine Effectiveness Against Severe Outcomes by Vaccine Type, Number of Doses, Time After Dose, and Case-Control Matching Ratios

			Matching ratio at 1:1[a]		Matching ratio at 1:2[b]	
			All registered patients	Hospitalized patients	All registered patients	Hospitalized patients
Number of doses	Vaccine type		VE against hospitalization/death (95% CI)	VE against death (95% CI)	VE against hospitalization/death (95% CI)	VE against death (95% CI)
One dose	CoronaVac		46.4 (43.0-49.6)	27.3 (18.8-35.0)	46.9 (43.9-49.7)	28.4 (20.5-35.4)
	BNT162b2		56.5 (49.6-62.5)	Undefined	57.5 (52.1-62.3)	29.4 (5.4-47.3)
Two doses	CoronaVac		67.9 (65.9-69.7)	49.3 (41.5-56.1)	69.9 (68.3-71.4)	51.0 (44.0-57.1)
	BNT162b2		75.1 (73.2-76.9)	57.2 (43.6-67.5)	76.2 (74.7-77.7)	55.9 (43.7-65.5)
Three doses	CoronaVac		84.5 (82.0-86.6)	71.1 (51.6-82.8)	85.3 (83.5-86.9)	74.4 (59.6-83.7)
	BNT162b2		84.6 (81.4-87.1)	71.6 (30.1-88.5)	84.2 (81.8-86.3)	76.0 (48.4-88.8)
	Mixing doses (two doses of CoronaVac + one dose of BNT162b2)		86.1 (81.7-89.4)	Undefined	86.9 (83.7-89.4)	64.8 (11.3-86.1)
Number of doses	Vaccine type	Time post dose (months)				
Two doses	CoronaVac	0-3	67.4 (64.2-70.3)	50.8 (39.4-60.1)	69.3 (66.6-71.7)	52.4 (42.3-60.7)
		4-6	68.3 (64.4-71.8)	45.8 (29.8-58.2)	70.3 (67.3-73.1)	44.0 (29.1-55.7)
		≥6	71.1 (67.4-74.3)	53.4 (32.6-67.8)	73.1 (70.4-75.6)	55.7 (38.4-68.2)
	BNT162b2	0-3	74.1 (69.4-78.1)	64.0 (41.3-77.9)	74.4 (70.4-77.8)	56.2 (33.2-71.3)
		4-6	73.1 (68.4-77.0)	58.7 (28.1-76.3)	73.3 (69.7-76.5)	57.9 (30.9-74.4)
		≥6	74.9 (71.6-77.8)	59.3 (29.7-76.5)	76.7 (74.3-78.9)	63.7 (42.4-77.2)
Three doses	CoronaVac	0-3	83.8 (80.8-86.4)	75.2 (53.7-86.8)	84.5 (82.3-86.5)	72.8 (54.1-83.9)
		4-6	85.9 (80.9-89.5)	63.3 (1.2-86.4)	87.4 (83.8-90.2)	82.1 (54.5-93.0)
	BNT162b2	0-3	84.2 (80.5-87.2)	76.9 (31.2-92.3)	83.8 (81.1-86.2)	71.3 (27.3-88.7)
		4-6	85.0 (75.0-91.0)	Undefined	84.3 (76.2-89.7)	76.7 (5.9-94.3)
	Mixing doses (two doses of CoronaVac + one dose of BNT162b2)	0-3	88.3 (83.2-91.9)	Undefined	87.1 (83.0-90.3)	Undefined
		4-6	83.7 (72.6-90.3)	Undefined	88.3 (81.8-92.5)	Undefined

Vaccination effectiveness was calculated as 1 minus the adjusted odds ratio which was obtained using a conditional logistic regression model adjusted with the covariates. Unvaccinated group was used as the reference level comparing with the vaccinated patients in a specific group (i.e., vaccine type and time post dose). Time post dose (months) is defined as the time since the last dose (second or third) to hospitalization or death for the vaccinated individuals. One thousand bootstrapped samples were used to construct the 95% CI around the mean estimates.

[a] In the analyses with matching ratio 1:1, the number of deaths/n of the unvaccinated group in all registered patients were 5812/25083, whereas the number of hospitalizations/n of the unvaccinated group in all registered patients were 15304/25083. The number of deaths/n of the unvaccinated group in the hospitalized patients were 4605/8439.

© 2023 Wei Y et al. *JAMA Network Open.*

[b] In the analyses with matching ratio 1:2, the number of deaths/n of the unvaccinated group in all registered patients were 5812/32909, whereas the number of hospitalizations/n of the unvaccinated group in all registered patients were 15304/32909. The number of deaths/n of the unvaccinated group in the hospitalized patients were 4605/11862.
Undefined: The estimates cannot be drawn due to insufficient size of outcome. VE=Vaccine effectiveness; CI=Confidence interval.

© 2023 Wei Y et al. *JAMA Network Open.*
