# Supplementary material for: Estimation of Vaccine Effectiveness of CoronaVac and BNT162b2 Against Severe Outcomes Over Time Among Patients With SARS-CoV-2 Omicron
Source: JAMA Netw Open. 2023 Feb 3;6(2):e2254777. doi: 10.1001/jamanetworkopen.2022.54777 (PMC9898822; doi:10.1001/jamanetworkopen.2022.54777)
Supplement: Supplement 2. — Data Sharing Statement [file jamanetwopen-e2254777-s002.pdf]

Data Sharing Statement

Wei. Estimation of Vaccine Effectiveness of CoronaVac and BNT162b2 Against Severe Outcomes Over Time Among Patients With SARS-CoV-2 Omicron. *JAMA Netw Open*. Published February 03, 2023. doi:10.1001/jamanetworkopen.2022.54777

Data
Data available: No

Additional Information
Explanation for why data not available: The sharing of data is restricted by the Department of Health, Hong Kong.
